# Coordinated expression of tyro3, axl, and mer receptors in macrophage ontogeny

**DOI:** 10.14800/macrophage.1261

**Published:** 2016-04-22

**Authors:** Anna Malawista, Xiaomei Wang, Mark Trentalange, Heather G. Allore, Ruth R. Montgomery

**Affiliations:** 1 Department of Internal Medicine, Yale University School of Medicine, New Haven, CT, 06519, USA; 2 Program on Human Translational Immunology, Yale University School of Medicine, New Haven, CT, 06519, USA

**Keywords:** Macrophage, Monocyte, TAM, Tyro3, Axl, Mer, Gas6, Protein S, TLR

## Abstract

The TAM receptors (Tyro3, Axl, and Mer) are a family of homologous receptor-tyrosine kinases that inhibit Toll-like receptor signaling to regulate downstream pathways and restore homeostasis. TAM triple mutant mice (Tyro3^−/−^, Axl^−/−^, Mer^−/−^) have elevated levels of pro-inflammatory cytokines and are prone to developing lymphoproliferative disorders and autoimmunity. Understanding differential expression of TAM receptors among human subjects is critical to harnessing this pathway for therapeutic interventions. We have quantified changes in TAM expression during the ontogeny of human macrophages using paired samples of monocytes and macrophages to take advantage of characteristic expression within an individual. No significant differences in levels of Tyro3 were found between monocytes and macrophages (flow cytometry: p=0.652, immunoblot: p=0.231, qPCR: p=0.389). Protein levels of Axl were reduced (flow cytometry: p=0.049, immunoblot: p<0.001) when monocytes matured to macrophages. No significant differences in the levels of Axl mRNA transcripts were found (qPCR: p=0.082), however, Tyro3 and Axl were proportionate. The most striking difference was upregulation of expression of Mer with both protein and mRNA being significantly increased when monocytes developed into macrophages (flow cytometry: p<0.001, immunoblot: p<0.001, qPCR: p=0.004). A fuller characterization of TAM receptor expression in macrophage ontogeny informs our understanding of their function and potential therapeutic interventions.

## Introduction

Pattern recognition receptors such as Toll-like receptors (TLRs) detect conserved molecular patterns on pathogens, including viruses, bacteria, and fungi ^[1–3]^. Upon detection of these common molecular epitopes, TLRs initiate immune pathways leading to the production of pro-inflammatory cytokines and other mediators of immunity. Inflammation and recruitment of immune cells is essential in response to infection; however, unregulated pro-inflammatory responses can result in tissue damage and lead to autoimmune disease ^[4]^. Thus, the activation of these receptors is tightly regulated to prevent excess inflammation and tissue damage ^[5]^.

The TAM receptors (Tyro3, Axl, and Mer) are a family of homologous receptor-tyrosine kinases that suppress TLRs and their downstream pathways to control excess stimulation and restore homeostatic balance ^[6, 7]^. TLR signaling induces TAM upregulation through the type I interferon receptor (IFNAR)-STAT1 pathway, which in turn suppresses the IFNAR-STAT1 pathway creating a self-regulating, negative feedback loop ^[7]^. The importance of the TAM regulatory mechanisms is evident in mice deficient for TAMs (Tyro3^−/−^, Axl^−/−^, Mer^−/−^), which have elevated levels of pro-inflammatory cytokines, including TNF-α and IL-6, and are prone to developing lymphoproliferative disorder and autoimmunity ^[6, 7]^.

The dysregulation of the TAM receptors has also been shown to play a role in cancer and tumorigenesis by reducing the efficacy of anti-tumor immune mechanisms and by decreasing tumor cell susceptibility to cytotoxic agents ^[8, 9]^. Thus TAMs are promising targets for novel therapeutic agents against cancer. Indeed, therapeutic drugs targeting the TAM pathways are actively under development, such as a protease inhibitor of Axl that has been shown to reduce metastatic burden in a mouse model of breast cancer, and a tyrosine kinase inhibitor that reduces the phosphorylation of Mer, which may target acute myeloid leukemia ^[10, 11]^.

The ontogeny of macrophage development follows a complex program from bone marrow precursors to circulating monocytes to tissue resident macrophages. Recent studies have revealed a range of macrophage phenotypes beyond pro-inflammatory and anti-inflammatory so called M1 and M2 and encompassing complexity of tissue-specific regulation of transcription factors and protein expression ^[12, 13]^. Levels of individual TAMs have been reported in murine models and show higher levels of Mer in macrophages from tissues ^[12, 14]^ and increased levels in myeloid cells from human intestines exposed to microbial products ^[15]^. However, variation among human subjects is considerable and a comprehensive measurement is lacking. Thus we have undertaken the current study using paired samples to take advantage of characteristic expression within an individual ^[16, 17]^ to elucidate the changes in expression of all three TAMs in the human monocyte maturation program.

## Materials and Methods

### Study Subjects

Heparinized blood was obtained from healthy donors (n=9) with written informed consent under an IRB protocol approved annually by the Human Investigations Committee of Yale University. At the time of enrollment self-reported data for all participants included demographic information. The blood donors were 44.4% female and 77.8% white reflecting the environment in our medical center. The average age was 26.4 (range 22–31) and our donors had no acute illness and were not on any antibiotics or nonsteroidal anti-inflammatory drugs within a month of enrollment and sample collection.

### Cell Preparation

Peripheral blood mononuclear cells (PBMCs) were isolated using Ficoll-Hypaque (GE Healthcare, NJ) as previously described ^[18]^. Monocytes were assessed immediately or following overnight culture. To derive macrophages, PBMCs were cultured for 6–8 days as described ^[18]^.

### Flow cytometry

Expression of TAMs was quantified in whole blood (200 μl/well) labeled in a 96 well plate in BD FACS Lysing solution (BD Biosciences, CA) as described ^[19]^. Following lysis of the red blood cells, cells were labeled for 30 min at 4^o^C protected from light with antibodies for surface lineage markers V500 conjugated CD45 (BD 560777), APC-Cy7-CD14 (BD 340585) and TAM receptors: PE anti-Axl (R & D Systems, MN FAB1541P), anti-Mer (R & D FAB8912P), and anti-Tyro (R & D FAB859P). Cells were washed with BD wash buffer and fixed in 1% paraformaldehyde. Data was acquired using an LSR II instrument (BD) and analyzed using FlowJo software (Tree Star, OR) ^[19]^.

### Immunoblot analysis

Total proteins were harvested using CelLytic M Cell Lysis buffer (Sigma, MO) containing protease inhibitor cocktail as described previously ^[19]^. Whole-cell lysates were electrophoresed on a 4–12% polyacrylamide gel (Invitrogen, CA) and processed for immunoblotting. Immunoblots were probed with anti-MerTK (B-1) (Santa Cruz Biotechnology, TX sc-365499), anti-Axl (R & D AF154), anti-Tyro3 (A-7) (Santa Cruz sc-166359), and anti-β-actin (Cell Signaling, MA 3700). Immunoblots were developed using a Western Lightning chemiluminescence kit (Pierce, IL), scanned, and densitometric analysis was performed with NIH ImageJ ^[19]^.

### Quantitative PCR (qPCR) analysis

Total RNA was harvested from cells using the RNeasy mini-kit according to the manufacturer’s instructions (Qiagen, CA). Primers and probes were from Applied Biosystems. Amplification was performed in a CFX96 Real-Time System (Bio-Rad, CA). All qPCR assays were done with one RNA isolation and two duplicate qPCR runs. Values for each gene were calculated from the accompanying standard curve in each qPCR plate. Each duplicate measurement was divided by the corresponding measurement for actin and then averaged.

### Statistical analysis

Descriptive statistics were generated for all variables. Distributions were checked for normality using a Shapiro-Wilk test. A paired t-test was used for normally distributed data, and non-normal data comparisons were analyzed using a Sign or Sign-rank test. Correlations were determined using Spearman’s rho. Statistical tests were 2-tailed, with P<0.05 considered significant. All analysis was conducted using SAS version 9.4 (SAS Institute Inc., Cary, NC, USA).

## Results and Discussion

Monocytes and macrophages differ in their localization and function ^[20, 21]^, and here we have quantified differential expression of TAM family receptors in paired samples from healthy subjects. Determining how TAM expression changes with macrophage ontogeny will support focused use of the TAM regulatory pathways as therapeutic targets.

**Figure 1:**
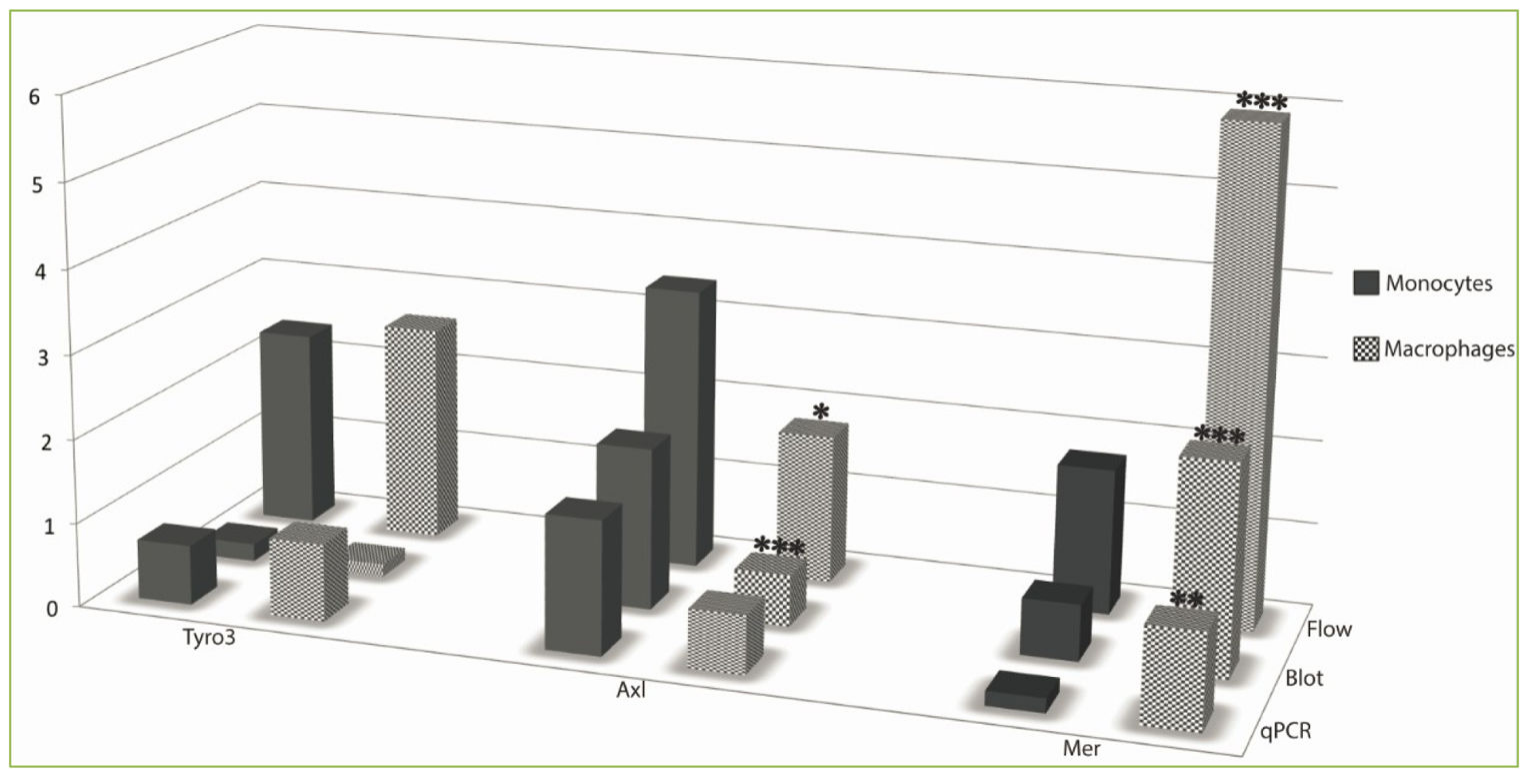
Levels of TAM receptors detected by flow cytometry, immunoblot, and qPCR TAM receptors (Tyro3, Axl, Mer) were quantified from paired samples of primary monocytes and macrophages from healthy donors (n=9). Data shown are means of levels of TAM receptors detected by flow cytometry with an antibody to each of the three TAMs and fluorescence levels were measured with FACS (% positive cells, scaled by 10); immunoblot: densitometry of TAM gene normalized to cellular actin (TAM/β-actin); and qPCR: mRNA was quantified by qPCR (SQ of TAM/SQ of β-actin). * indicates significant comparison with paired monocytes, *P < 0.05; **, P < 0.01; ***, P < 0.001.

We quantified levels of Tyro3 in paired monocyte and macrophage samples from healthy donors. Total expression of Tyro3 quantified by flow cytometry was detected on 23.7% of monocytes and was not significantly different between monocytes and macrophages (p=0.652) (Fig. 1 & S1A). When Tyro3 protein levels were quantified by immunoblot, we detected very low expression in both monocytes and macrophages with no significant differences between the groups (p=0.231) (Fig. 1 & S1B). Similarly, levels of Tyro3 mRNA were not significantly different (p=0.389) (Fig. 1 & S1C), indicating that expression of Tyro3 is relatively low across the two cell stages.

Significant downregulation of total protein expression of the Axl receptor was detected by both flow cytometry (p= 0.049) and immunoblot (p<0.001) and diminished by 2–3 fold on monocyte maturation into macrophages (Fig. 1 & S1D–E). The levels of Axl RNA transcripts appeared somewhat lower in macrophages than monocytes but did not reach statistical significance (p= 0.082) in (Fig. 1 & S1F). The significant reduction detected at the protein level suggests that reduction of Axl expression in macrophages may occur through post transcriptional or translational modifications, such as shedding of this receptor to the soluble form of Axl (sAxl), as has been noted previously ^[22]^.

Notably, the RNA expression levels of Tyro3 and Axl are correlated in monocytes. A significant positive correlation between the transcript levels of Axl and Tyro3 was detected (r=0.850, P=0.006) between the transcriptional expression of Axl and that of Tyro3 (Fig. S2) suggesting shared regulatory processes.

We quantified levels of protein and mRNA expression of Mer as monocytes develop into macrophages. Total Mer protein was found to be significantly upregulated in macrophages as compared to monocytes by both flow cytometry (p<0.001) and immunoblot (p<0.001) (Fig. 1 & S1F–G). In addition, the levels of Mer mRNA were significantly higher in macrophages than monocytes (p=0.004) (Fig. 1 & S1I). This finding suggests that the upregulation of Mer that occurs in development of macrophages may include regulation of transcription, translation, or both.

The different expression levels of TAMs in monocytes and macrophages are relevant to their activation by their soluble ligands, Protein S and growth arrest-specific gene 6 (Gas6) ^[23]^. The abundance of these two ligands in circulation differs, with significantly higher concentration of Protein S in the blood (~300 nM), whereas Gas6 levels in the blood are relatively low (~0.02–0.2 nM) ^[24]^. Thus, monocytes in circulation are constantly exposed to Protein S, although it is only activating for apoptotic engulfment following oxidation ^[25]^. Gas6 has been shown to bind to all three TAM receptors, while Protein S preferentially activates Tyro3 and Mer ^[26, 27]^. High levels of Mer in macrophages suggest a key role for Mer in the tissue, and low levels of Mer in monocytes may reflect a regulatory mechanism for Mer activation in high concentrations of ligand, Protein S, such as are found in the blood ^[24]^. However, as Mer uses both Gas6 and protein S as ligands ^[24, 28]^, both circulating monocyte and tissue macrophages would be expected to be exposed to a high level of activating ligand.

The exposure of Axl to its ligand, Gas6, and the potential to activate this receptor differs greatly between monocytes and macrophages. Unbound Gas6 levels are very low in circulation and Gas6 is located almost exclusively in the tissues ^[29]^. Circulation of the sAxl-Gas6 complex is indicative of inflammation ^[28]^. Changes in expression of Axl by the cell may indicate a role for Gas 6 regulation of monocytes in blood where expression of the receptor is relatively high and that of the ligand is low; whereas, the lower expression of Axl in macrophages could help to regulate Axl activation in a tissue environment with higher concentration of Gas6. It is also important to note that, although both Mer and Axl mediate phagocytosis, Mer has been reported to mediate homeostatic phagocytosis of apoptotic cells and Axl is the key receptor for initiation of phagocytosis at sites of inflammation initiated by infection or trauma ^[26]^.

Unregulated TLRs have been shown to be involved in several autoimmune and inflammatory diseases ^[6, 7]^. TAMs, as natural TLR inhibitors, provide a valuable strategy against inflammatory disorders for a wide variety of applications, in cancer and autoimmunity studies. Human subjects vary widely in genetic background and environmental exposures and variability between donors is well documented ^[16, 17]^. We have used paired samples to reduce variation evident among human subjects and characterize TAM expression in macrophage ontogeny. Even with a small sample size, taking advantage of unique steady state of individuals revealed changes in expression relevant to therapeutic intervention.

## Supplementary Material



## Abbreviations

TAM: Tyro3, Axl, and Mer
TLR: Toll-like receptor
Gas6: growth arrest-specific gene 6
PBMCs: Peripheral blood mononuclear cells
