# Supplementary material for: Coordinated expression of tyro3, axl, and mer receptors in macrophage ontogeny
Source: Macrophage (Houst). Author manuscript; Available in PMC 2016 Sep 28. (PMC5040214; doi:10.14800/macrophage.1261)
Supplement: Supplementary file 1 [file NIHMS781784-supplement-supplement_1.pdf]

Supplements

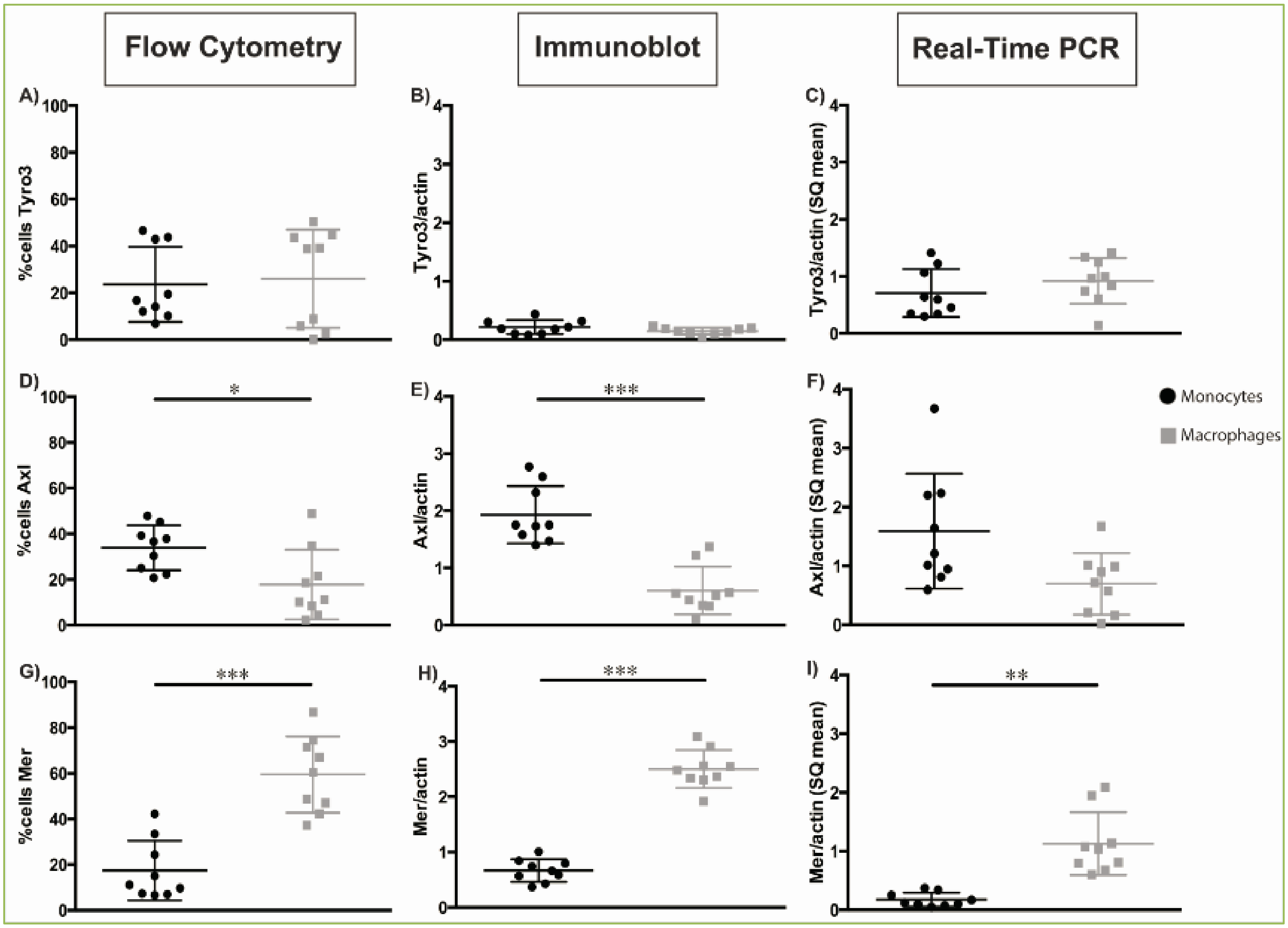

Figure S1. Raw data of levels of TAM receptors detected by flow cytometry, immunoblot, and qPCR. TAM receptors (Tyro3, Axl, Mer) were quantified from paired samples of primary monocytes and macrophages from healthy donors; raw data are shown with means and standard deviations (n=9). P-values correspond to T-tests or appropriate non-parametric comparisons (*, P < 0.05; **, P < 0.01; ***, P < 0.001). A, D, G) Human primary monocytes and macrophages from healthy donors were labeled with antibody to each of the three TAMs and fluorescence levels were measured with FACS percent positive cells. B, E, H) Monocytes and macrophages protein immunoblot were probed for TAMs and normalized to actin; densitometry analysis was performed to produce quantitative measures of protein levels. C, F, I). mRNA was harvested and cDNA was synthesized from primary monocytes and macrophages of healthy donor for qPCR (gene/β-actin).

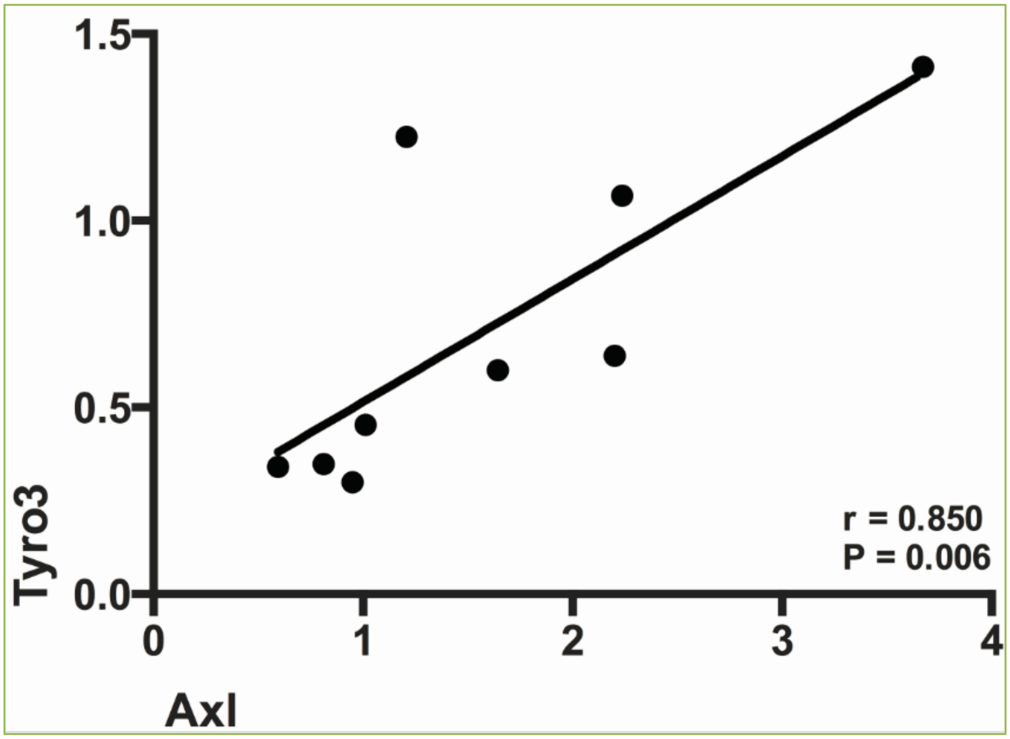

Figure S2. **Axl and Tyro3 RNA transcripts from monocytes are significantly correlated with one another:** Transcript levels of Axl and Tyro3 were graphed on a scatter plot and Spearman's Rho was calculated (shown on graph) indicating that there is a significant positive correlation between monocyte expression of Axl and Tyro3 RNA transcripts.
